# Five New Species of *Orthosinus* Motschulsky, 1863 from China—Molecular Evidence for Two Species (Coleoptera, Curculionidae, Dryophthorinae) †

**DOI:** 10.3390/insects17070691

**Published:** 2026-07-02

**Authors:** Heyu Lü, Runzhi Zhang

**Affiliations:** 1 Key Laboratory of Animal Biodiversity Conservation and Integrated Pest Management, Institute of Zoology, Chinese Academy of Sciences, No. 1 Beichen West Road, Chaoyang District, Beijing 100101, China; lvheyu23@ioz.ac.cn; 2 College of Life Science, University of Chinese Academy of Sciences, Beijing 100049, China

**Keywords:** DNA barcode, identification key, Stromboscerini, morphology, taxonomy, weevils

## Abstract

Five new species of *Orthosinus* Motschulsky, 1863 from China are described: *O. borisi* sp. nov. (♂♀) and *O. urceolatus* sp. nov. (♂♀) from Xizang Autonomous Region, *O. diaoluoshanensis* sp. nov. (♂) from Hainan Province, *O. sulcatus* sp. nov. (♂♀) from Sichuan Province and *O. tengchongensis* sp. nov. (♂) from Yunnan Province. Detailed morphological descriptions, diagnostic illustrations, and habitus photographs are provided for each species. An identification key and a distribution map for all known Chinese species of *Orthosinus* are also provided. With these additions, the number of known Chinese species of *Orthosinus* is increased from two to seven. Molecular phylogenetic and species delimitation analyses of the *COI* gene were additionally performed for two of the newly described species, *O. urceolatus* sp. nov. and *O. sulcatus* sp. nov., along with other congeneric and outgroup specimens.

## Simple Summary

Small weevils of the tribe Stromboscerini inhabit leaf litter in tropical and subtropical forests. Southern China is considered a diversity hotspot for this group, but many species are still poorly known. The genus *Orthosinus* belongs to this tribe and was previously represented in China by only two species. Based on specimens discovered in museum collections, we describe five new species of *Orthosinus* from Xizang, Hainan, Sichuan, and Yunnan, significantly expanding the known distribution and species richness of the genus in China. Detailed morphological descriptions, diagnostic photographs, an identification key, and a distribution map are provided. For two of the new species, DNA barcode sequences and phylogenetic analyses also confirm their distinct species status. This study raises the number of Chinese *Orthosinus* species to seven and provides a foundation for future work on this poorly understood genus.

## 1. Introduction

The weevil genus *Orthosinus* Motschulsky, 1863 (type species *O. velatus* Lacordaire, 1866) belongs to the tribe Stromboscerini, subfamily Dryophthorinae [1]. The tribe Stromboscerini currently comprises 14 genera worldwide, with eight genera and 16 species recorded from China. Stromboscerini is a relatively small tribe whose members primarily inhabit forest floor litter and are often associated with Pinaceae (including *Orthosinus* from *Abies* forests) [2,3,4]. The keys to genera of Stromboscerini provided by Morimoto remain the principal taxonomic framework for the tribe and were used in the present study to assess generic placement [5,6]. All five new species described herein conform to the diagnostic characters of *Orthosinus* and readily key to this genus. Following a recent re-examination by Lü and Zhang [7], the morphologically similar genus *Xerodermus* was treated as a junior synonym of *Orthosinus*.

The genus *Orthosinus* currently comprises 15 species distributed across China, India, Japan, Java, Laos, Myanmar, Nepal, and Sri Lanka [7]. The Chinese fauna of *Orthosinus* has long been poorly documented. The earliest record traces back to Voss [8], who described *O. foveatus* Voss, 1953 from Fujian Province. More recently, Lü and Zhang [7] added *O. medogensis* Lü & Zhang, 2026 from Mêdog County, Xizang Autonomous Region, bringing the total to only two recorded Chinese species prior to this study.

Continued examination of weevil specimens from various Chinese localities has now revealed five additional undescribed species. These are *O. borisi* sp. nov. and *O. urceolatus* sp. nov. from Xizang, *O. diaoluoshanensis* sp. nov. from Hainan, *O. sulcatus* sp. nov. from Sichuan, and *O. tengchongensis* sp. nov. from Yunnan. These discoveries significantly expand the distribution and documented diversity of the genus in China. The new species from Hainan Island is particularly noteworthy, as it represents the first record of *Orthosinus* from a Chinese offshore island.

This paper provides detailed morphological descriptions, diagnostic illustrations, habitus photographs, an identification key, and a distribution map for all known Chinese *Orthosinus* species. For two species (*O. urceolatus* sp. nov. and *O. sulcatus* sp. nov.), a COI-based molecular assessment is additionally conducted using Maximum Likelihood phylogenetic reconstruction and two species delimitation methods (ABGD and jMOTU). All analyses consistently support their distinct taxonomic status, complementing the morphological evidence. This integrative approach provides a basis for future systematic work on this poorly understood group.

## 2. Materials and Methods

### 2.1. Taxon Sampling

The specimens examined in this study were discovered during a search of the museum’s collections. All specimens are deposited in the Institute of Zoology, Chinese Academy of Sciences, Beijing, China (IZCAS).

### 2.2. Specimen Examination

All morphological observations, measurements, and dissections were conducted using a Nikon SMZ18 stereomicroscope (Nikon, Minato City, Japan) with coaxial LED illumination. All dissections involved removing the abdomen or posterior of the relevant specimen, placing the sectioned material in 10% KOH solution and heating in a boiling water bath for between 5 and 15 min depending on the condition of the specimen. All soft tissues were removed, leaving the sclerotized parts of the genitalia. Specimens were imaged using a Visionary Digital LK Lab System (WeMacro, Shanghai, China) equipped with a Sony A7RM5 camera and 5×, 10×, and 20× Mitutoyo objective lenses (Sony, Tokyo, Japan). Multifocal image stacking was processed in Helicon Focus v. 7.5.4 Pro. All image plates were assembled and adjusted in Adobe Photoshop CC 2019. The species distribution map was generated in QGIS v. 3.28.15, integrating georeferenced locality data over a high-resolution basemap sourced from Tianditu (https://www.tianditu.gov.cn/, accessed on 10 December 2025).

Measurements follow Lü and Zhang [9], with the abbreviations: body length (Bl), rostrum length (Rl), rostrum width (Rw), pronotum length (Pl), pronotum width (Pw), elytra length (El), and elytra width (Ew = body width).

Terminology for general morphological structures primarily follows the online glossary of weevil characters proposed in the International Weevil Community Website (https://www.curculionoidea.org/glossary, accessed on 20 April 2026). Male genitalia terminology follows Oberprieler et al. [10], while terminology for certain leg structure follows Girón and Chamorro [11].

### 2.3. DNA Extraction, PCR Amplification and Sequencing

Total DNA was extracted using the Qiagen DNeasy Blood & Tissue Kit (Qiagen Sciences, Inc., Germantown, MD, USA), and the final elution volume was 60 μL. The extracted total DNA was stored at −20 °C in a freezer for subsequent amplification and sequencing. Cytochrome c oxidase subunit 1 (*COI*) sequences of new species were amplified using universal primers LCO1490 (forward) and HCO2198 (reverse) [12]. The PCR reaction protocol: initial denaturation at 95 °C for 3 min; 38 cycles of denaturation at 95 °C for 30 s, annealing at 45 °C for 40 s, and elongation at 72 °C for 7 min; and final extension at 72 °C for 7 min. The 25 μL PCR reactions included 12.5 μL of 2 × Tag Master Mix (Tiangen, Beijing, China), 1.0 μL of each forward and reverse 10 μM primer, 2 μL of genomic DNA, and 8.5 μL of double-distilled H_2_O. The PCR products were sent to Tsingke Biotechnology Co., Ltd. (Beijing, China) for sequencing.

### 2.4. Data Analyses

Using the Mesquite v. 3.61 [13] package Chromaseq [14], sequence chromatograms were checked, assembled, and manually calibrated. Sequences were uploaded to the National Center for Biotechnology Information (NCBI) and GenBank accession numbers are listed in Table 1.

Phylogenetic analysis was based on the *COI* gene fragment using maximum likelihood (ML). A best-fit model was tested according to the corrected Akaike’s Information Criterion (AICc) using ModelFinder (included in IQ-TREE) with PhyloSuite v. 1.2.2 [15]. The ML tree search was performed in IQ-TREE v. 1.6.8 [16]. The ML tree was inferred using an edge-linked partition model for 5000 ultrafast bootstraps (1000 replicates) [17]. Support for each node is represented by ultrafast bootstrap values. The phylogenetic trees were visualized and illustrated using tvBOT (https://www.chiplot.online/tvbot.html (accessed on 20 April 2026) [18]). Species delimitation employed two distance-based methods: Automatic barcode gap discovery (ABGD; [19]) and java molecular operational taxonomic units (jMOTU; [20]). ABGD was conducted based on the Kimura (K80) TS/TV 2.0 model with prior value of intraspecific divergence between 0.001 and 0.1, 10 recursive steps, and relative gap width (X) of 0.5. For jMOTU analysis, the low blast identity filter was set to 97% and the percentage of minimum sequence length was set to 95.

**Table 1:** Voucher specimen and sequences information.

Species	Voucher Code	Sex	GenBank Accession Number	Collection Localities	Source
*Orthosinus urceolatus* sp. nov.	HYL004	Female	PZ092820	China, Xizang	This study
*Orthosinus sulcatus* sp. nov.	HYL005	Female	PZ092819	China, Sichuan	This study
*Orthosinus* sp.	GL15	-	HQ986782	China, Yunnan	Grebennikov [21]
*Orthosinus* sp.	GL15	-	HQ986783	China, Yunnan	Grebennikov [21]
*Orthosinus* sp.	CN06	-	MG968917	China, Yunnan	Grebennikov [21]
*Tasactes* sp.	CN02	-	HQ987041	China, Yunnan	Grebennikov [21]
*Tasactes* sp.	EM15	-	HQ986820	China, Sichuan	Grebennikov [21]
*Tasactes* sp.	GN03	-	MG968925	China, Sichuan	Grebennikov [21]
*Allaeotes* sp.	TD02	-	MG968948	Vietnam	Grebennikov [21]
*Allaeotes* sp.	TD06	-	MG968939	Vietnam	Grebennikov [21]

## 3. Results

### 3.1. COI Sequence Analysis

**Figure 1:**
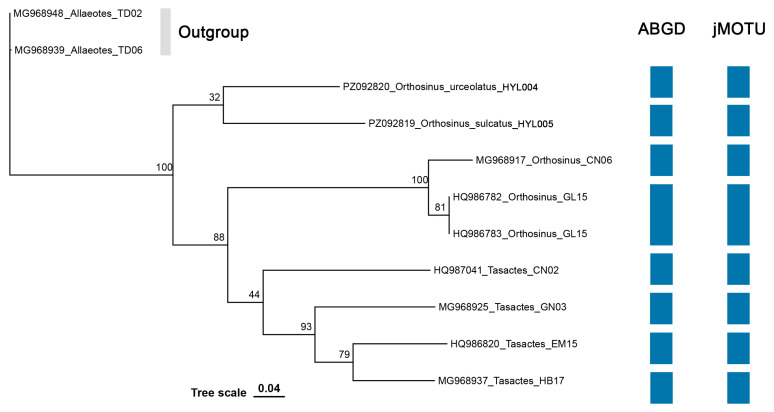
Maximum likelihood tree using the 11 *COI* sequences. Bootstrap support values are shown above the nodes. The results of delimitation analyses are displayed with the vertical bars corresponding to putative species (MOTUs) inferred by ABGD and jMOTU methods.

In the Maximum Likelihood tree inferred from 11 *COI* sequences, *Orthosinus* and *Tasactes* were recovered as two well-supported sister clades (Figure 1). Within *Orthosinus*, *O. urceolatus* sp. nov. and *O. sulcatus* sp. nov. formed a moderately supported clade (bootstrap = 32), likely reflecting the currently limited taxon sampling, sister to a strongly supported clade (bootstrap = 100) containing *O.* sp. (CN06) and the two identical sequences of *O.* sp. (GL15). The two species delimitation methods, ABGD and jMOTU, produced fully congruent results, dividing the ingroup sequences into eight primary molecular operational taxonomic units (MOTUs). In *Orthosinus*, each of the two new species was delimited as a distinct MOTU, whereas the two sequences of *O.* sp. (GL15) were merged into a single MOTU. *O.* sp. (CN06) was recovered as a separate MOTU. These results support the recognition of the two new species and highlight genetic distinction among the sampled *Orthosinus* specimens.

### 3.2. Taxonomy

Genus *Orthosinus* Motschulsky, 1863
Type species: *Orthosinus velatus* Lacordaire, 1866 (designated by Alonso-Zarazaga & Lyal [22])
Diagnosis. See Lü and Zhang [7]
Distribution. China, India, Japan, Indonesia (Java), Laos, Myanmar, Nepal, Sri Lanka.

#### 3.2.1. *Orthosinus borisi* Lü & Zhang sp. nov.

**Figure 2:**
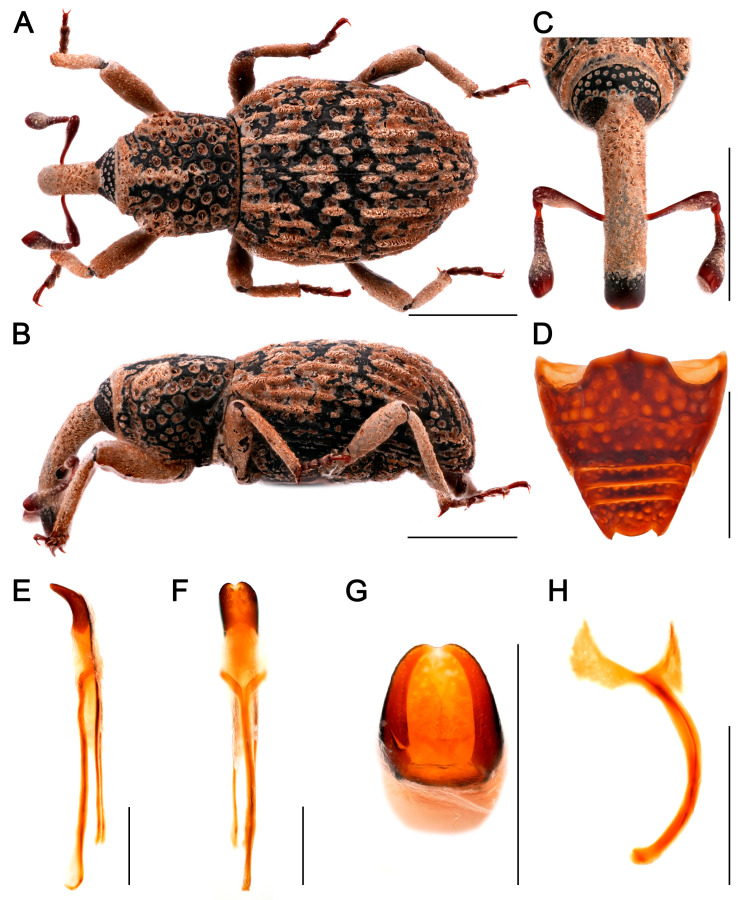
*Orthosinus borisi* sp. nov., holotype. (**A**) Dorsal habitus; (**B**) Lateral habitus; (**C**) Head, dorsal view; (**D**) Ventrites, ventral view; (**E**) Penis, lateral view; (**F**) Penis, ventral view; (**G**) Penis at apex, showing details of the pedon; (**H**) Spiculum gastrale. Scale bars: 1 mm (**A**–**D**); 0.5 mm (**E**–**H**).

**Figure 3:**
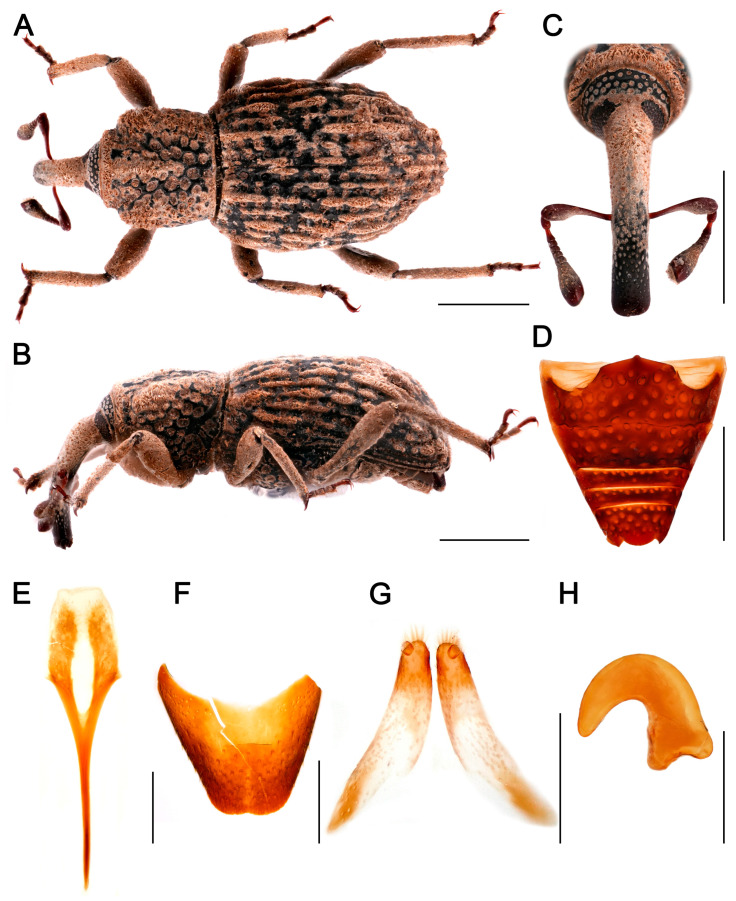
*Orthosinus borisi* sp. nov., paratype female (IOZ(E) 1965782). (**A**) Dorsal habitus; (**B**) Lateral habitus; (**C**) Head, dorsal view; (**D**) Ventrites, ventral view; (**E**) 8th sternite; (**F**) 8th tergite; (**G**) Ovipositor; (**H**) Spermatheca. Scale bars: 1 mm (**A**–**D**); 0.25 mm (**E**–**H**).

urn:lsid:zoobank.org:act:A1CF48CB-1DBE-498C-919E-11E9841157E3
Figure 2 and Figure 3
Material examined. Holotype: 1♂, China, Xizang, Linzhi City, Bomi County, G318 (Tongmai Bridge), 30.0975° N, 95.0660° E, elev. 1988.02 m, 20/VII/2019, Run Zhou and Zhuo Ma leg. IOZ(E) 1965786. Paratypes: 11♂♂13♀♀, same data as holotype, IOZ(E) 1965782–1965784, IOZ(E) 1965787–1965790, IOZ(E) 1965807–1965809, IOZ(E) 1965811–1965819, IOZ(E) 1965821, IOZ(E) 1965822, IOZ(E) 1965824, IOZ(E) 1965826, IOZ(E) 1965853. 1♀, Xizang, Linzhi City, Bomi County, Tongmai Bridge (with lights under the bridge), 30.09647° N, 95.06602° E, elev. 2001 m, 30/VII/2018, collector unknown. IOZ(E) 1965807. 1♂, Xizang, Linzhi City, Bayi District, Pailong Township, Polong Gou, 30.02421° N, 95.00770° E, elev. 1923 m, 03/VIII/2018, Run Zhou leg. IOZ(E) 1965810. 1♀, Xizang, Linzhi City, Bomi County, Yigong Township (20 km along Provincial Highway S305), 30.17466° N, 94.93364° E, elev. 2323 m, 05/VIII/2018, Run Zhou leg. IOZ(E) 1965796. 4♂♂5♀♀, Xizang, Linzhi City, Bomi County, G318 (Tongmai Bridge), 30.0975° N, 95.0660° E, elev. 1988.02 m, 18/VII/2019, Run Zhou and Zhuo Ma leg. IOZ(E) 1965795, IOZ(E) 1965797, IOZ(E) 1965798, IOZ(E) 1965800–1965805. 1♀, Mêdog County, near Xirong Gorge, 62 km along the Mêdog Highway, 29.2311° N, 95.1400° E, elev. 735.88 m, 26/VII/2019, Run Zhou and Zhuo Ma leg. IOZ(E) 1965806.
Type locality. G318 (Tongmai Bridge), Bomi County, Linzhi City, Xizang Autonomous Region, China.
**Comparative diagnosis.** *O. borisi* sp. nov. is most similar to *O. tuberculatus* Legalov, 2021 [3] but differs in the following characters: (i) smaller body (Bl: 3.50–4.30 mm vs. 5.10–5.80 mm in *O. tuberculatus*); (ii) antennal scape and club more slender: scape 5.0 times as long as wide, club 1.82 times as long as wide (vs. scape 4.0 times, club 1.5 times in *O. tuberculatus*); (iii) pronotal sides subparallel, with dense, coarse punctures (vs. sides rounded, punctures sparse, with weak median carina in *O. tuberculatus*); (iv) elytral interstriae as wide as striae (vs. slightly wider than striae in *O. tuberculatus*). *O. borisi* sp. nov. is geographically closest to *O. medogensis* and *O. urceolatus* sp. nov. but can be distinguished by the following characters: rostrum more slender (Rl: 1.20–1.50 mm vs. 1.05–1.40 mm in the latter two); pronotum with dense pilose pustules (vs. absent in *O. medogensis* and *O. urceolatus* sp. nov.); pilose pustules on elytral interstriae broader; and differences in male penis, female 8th sternite, and spermatheca (Figures 2E–G and 3E,H vs. Lü & Zhang [7]: Figures 1I–K, 2A–D and 3G,J for *O. medogensis*; and vs. Figures 8E–G and 9E,H for *O. urceolatus* sp. nov.).
**Description. *Coloration*** (Figure 2A,C). Body entirely black; rostral apex, antennae, and tarsomeres reddish brown.
***Head*** (Figure 2C). Subglobular, with dense, small punctures, with a shallow, transverse sulcus between frons and rostrum; eyes elongate-oval, widely separated ventrally; rostrum elongate (Rl/Rw 4.00), longer than pronotum (Rl/Pl 1.04), evenly curved in lateral view, base thick, with dense, short pubescence and coarse punctures from base to apical one-fourth; antennae inserted anterior to rostral mid-length; scape long (l/w 5.00), not reaching eyes, gradually broadening from base to apex, apical one-third markedly widened; funicular segments 1 and 2 elongate, segment 1 longer than wide, segment 2 funnel-shaped, length equal to combined length of segments 3 + 4, segments 3–6 transverse; club spindle-shaped (l/w 1.82).
***Pronotum*** (Figure 2A,B). Shorter than wide (Pl/Pw 0.95), widest at apical one-third, apical one-sixth distinctly constricted, sides rounded, gradually narrowed from apical one-third to base; disc slightly convex in lateral view, with dense, coarse punctures, distance between punctures smaller than puncture diameter, punctures sparser and smaller on constricted area than elsewhere; densely covered with short pubescence, apical with pilose pustules; postocular lobes absent.
***Scutellum*.** Small, subtriangular.
***Elytra*** (Figure 2A,B). Longer than wide (El/Ew 1.22), widest at basal one-fourth, apical one-seventh distinctly constricted, sides rounded; disc nearly flat in lateral view; interstriae slightly elevated, subequal in width, with dense, short pubescence; interstriae 1–8 bearing interrupted longitudinal pilose pustules, pustule width subequal to interstrial width; striae deep, as wide as interstriae; punctures rounded, distance between punctures subequal to a puncture diameter, punctures with dense, short pubescence.
***Abdomen*** (Figure 2D). Abdominal ventrites densely covered with coarse punctures, punctures on 3rd and 4th ventrites sparser than on others, punctures at margins larger than those at middle; 2nd ventrite with anterior margin slightly convex at middle, posterior margins of ventrites 2–4 rectilinear; 2nd ventrite 0.7 times length of 1st ventrite, 3rd ventrite as long as 4th ventrite, 5th ventrite 2.5 times as wide as long, deeply emarginate apically.
***Legs*** (Figure 2A,B). Densely covered with short pubescence; femora and tibiae with punctures; procoxae subconical, contiguous; mesocoxae narrowly separated; profemur more robust than mesofemur and metafemur; femora unarmed; profemur 4.0 times as long as wide; tibiae bearing single, long uncus; tarsi long, tarsomeres 1–3 obconical, ventrally with dense erect setae, onychium elongate; claws free, divergent.
***Male genitalia*** (Figure 2E–H). Pedon 0.3 times length of temones, gradually broadening from base to apical one-third, then narrowed apically, curved in lateral view, sides rounded, base symmetrical, apex thin in lateral view; temones slender, slightly curved; manubrium long, as long as temones, slightly curved from base to apex, approximately 2.0 times as wide as temones; spiculum gastrale robust, evenly curved; basal plate bifurcate, basal arms opposed, upper part of each basal arm approximately triangular, apices acute.
***Female*** (Figure 3A–H). Body larger than in male; rostrum longer, smooth and shining; antennae inserted slightly anterior to middle of rostrum; punctures on pronotum larger than in male. 8th sternite with apodeme 1.4 times length of lamina; lamina bifurcate at base, sides slightly curved, apex with setae; 8th tergite 0.9 times as long as wide, posterior margin nearly smooth, mesally deeply emarginate; surface gradually more coarsely punctate towards apex, from apical one-half to apex densely setose. Gonocoxites cylindrical, apices with dense, long setae; styli short, cylindrical, width approximately one-half width of gonocoxite apices, apices with setae; spermatheca with curved, apically rounded cornu; corpus very robust; ramus and collum weakly developed; other characters without distinct differences from male.
**Measurement (mm).** Holotype. Bl: 3.55. Rl: 1.20, Rw: 0.30. Pl: 1.15, Pw: 1.20. El: 2.20, Ew: 1.80. Male paratypes. (*n* = 16): Bl: 3.50–4.30 (3.77). Rl: 1.23–1.50 (1.31), Rw: 0.30–0.35 (0.31). Pl: 1.10–1.40 (1.22), Pw: 1.10–1.40 (1.22). El: 2.20–2.55 (2.34), Ew: 1.70–2.20 (1.88). Female paratypes. (*n* = 21): Bl: 3.50–4.20 (3.81). Rl: 1.20–1.42 (1.34), Rw: 0.30–0.38 (0.34). Pl: 1.00–1.33 (1.24), Pw: 1.14–1.40 (1.26). El: 2.10–2.70 (2.36), Ew: 1.65–2.10 (1.88).
**Distribution.** Bayi District, Bomi County, and Medog County, Linzhi City, Xizang Autonomous Region, China (Figure 10).
**Etymology.** The specific epithet is in honor of Dr. Boris A. Korotyaev, the Russian curculionidologist who made great contributions on the taxonomy of Curculionidae and helped us in many ways.

#### 3.2.2. *Orthosinus diaoluoshanensis* Lü & Zhang sp. nov.

**Figure 4:**
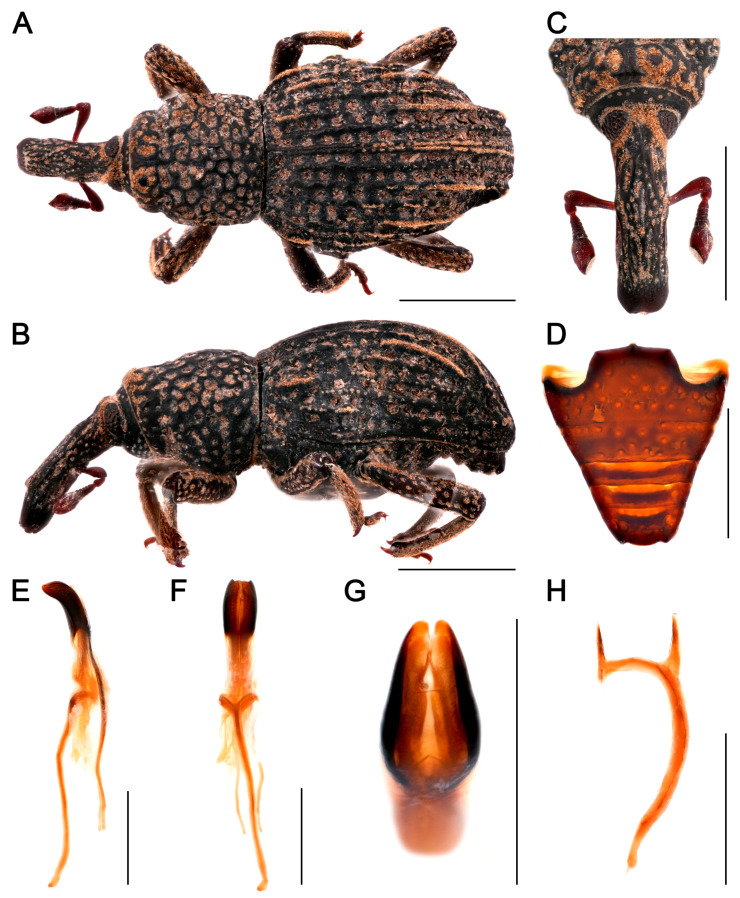
*Orthosinus diaoluoshanensis* sp. nov., holotype. (**A**) Dorsal habitus; (**B**) Lateral habitus; (**C**) Head, dorsal view; (**D**) Ventrites, ventral view; (**E**) Penis, lateral view; (**F**) Penis, ventral view; (**G**) Penis at apex, showing details of the pedon; (**H**) Spiculum gastrale. Scale bars: 1 mm (**A**–**D**); 0.5 mm (**E**–**H**).

urn:lsid:zoobank.org:act:4DDD74CF-2040-485D-A5C2-50D72B8F1766
Figure 4
Material examined. Holotype: 1♂, China, Hainan Province, Lingshui County, Diao Luo Mountain, elev. 920 m, 04/V/2007, Zongyi Zhao leg. IOZ(E) 1854450.
Type locality. Diaoluo Mountain, Lingshui County, Hainan Province, China.
**Comparative diagnosis.** *O. diaoluoshanensis* sp. nov. morphologically is most similar to *O. foveatus*, but distinguished by: (i) larger body (Bl: 4.40 mm vs. 3.50 mm in *O. foveatus*); (ii) rostrum shorter than pronotum (vs. subequal in *O. foveatus*); (iii) antennal club stouter (l/w 1.67 vs. almost 2.00 in *O. foveatus*); (iv) pronotum as long as wide, widest at middle, disc with coarse punctures not forming rugae (vs. longer than wide, widest at apical one-third, punctures forming longitudinal rugae medially in *O. foveatus*).
**Description. *Coloration*** (Figure 4A,B). Body entirely black; antennae and tarsomeres reddish brown.
***Head*** (Figure 4C). Subglobular, with dense, small punctures, with a shallow, transverse sulcus between frons and rostrum; eyes oval, widely separated ventrally; rostrum elongate (Rl/Rw 3.16), shorter than pronotum (Rl/Pl 0.95), evenly curved in lateral view, base convex, with dense, short pubescence and coarse punctures from base to apical one-fourth, dorsally with short, indistinct longitudinal carina; antennae inserted posterior to rostral mid-length; scape long (l/w 3.82), not reaching eyes, gradually broadening from base to apex; funicular segments 1 and 2 elongate, segment 1 longer than wide, segment 2 funnel-shaped, length equal to combined length of segments 3 + 4, segments 3–6 transverse; club spindle-shaped (l/w 1.60).
***Pronotum*** (Figure 4A,B). As long as wide (Pl/Pw 1.00), widest at middle, apical one-fifth distinctly constricted, sides subparallel; disc distinctly convex in lateral view, with dense, coarse punctures, distance between punctures much smaller than puncture diameter, punctures sparser and smaller on constricted area than elsewhere; densely covered with short pubescence, anterior margin without pilose pustules; postocular lobes absent.
***Scutellum*.** Small, elongate-oval.
***Elytra*** (Figure 4A,B). Longer than wide (El/Ew 1.19), widest at basal one-third, with small arcuate tubercles laterally, distinctly constricted at apical one-fourth, sides rounded; disc distinctly convex in lateral view; interstriae elevated, subequal in width, with dense, short pubescence; interstriae 1, 3 and 5 have more continuous pubescence, from apical one-third to apex denser than on other interstriae; striae deep, wider than interstriae; punctures rounded, distance between punctures smaller than a puncture diameter, punctures with dense, short pubescence.
***Abdomen*** (Figure 4D). Abdominal ventrites covered with coarse punctures, punctures on 3rd and 4th ventrites sparser than on others, punctures at margins slightly larger than those at middle; 2nd ventrite with anterior margin nearly rectilinear at middle, posterior margins of ventrites 2–4 rectilinear; 2nd ventrite 0.5 times length of 1st ventrite, 3rd ventrite as long as 4th ventrite, 5th ventrite 2.4 times as wide as long, deeply emarginate apically.
***Legs*** (Figure 4A,B). Densely covered with short pubescence; femora and tibiae with punctures; procoxae subconical, contiguous; mesocoxae narrowly separated; profemur more robust than mesofemur and metafemur; femora unarmed; profemur 3.3 times as long as wide; tibiae bearing single, long uncus; tarsi long, tarsomeres 1–3 obconical, ventrally with dense erect setae, onychium elongate; claws free, divergent.
***Male genitalia*** (Figure 4E–H). Pedon 0.3 times length of temones, gradually broadening from base to middle, then narrowed apically, curved in lateral view, sides rounded, base symmetrical, apex thin in lateral view; temones slender, distinctly curved; manubrium long, slightly curved, as long as temones, approximately 1.8 times as wide as temones; spiculum gastrale robust, evenly curved, widest at middle; basal plate bifurcate, basal arms slender, opposed, apices acute.
**Female.** unknown.
**Measurement (mm).** Holotype. Bl: 3.60. Rl: 1.14, Rw: 0.40. Pl: 1.20, Pw: 1.20. El: 2.20, Ew: 1.85.
**Distribution.** Known only from the type locality in Hainan Province, China (Figure 10).
**Etymology.** The new species is named after its type locality, Diaoluo Mountain. Adjective, variable.

#### 3.2.3. *Orthosinus sulcatus* Lü & Zhang sp. nov.

**Figure 5:**
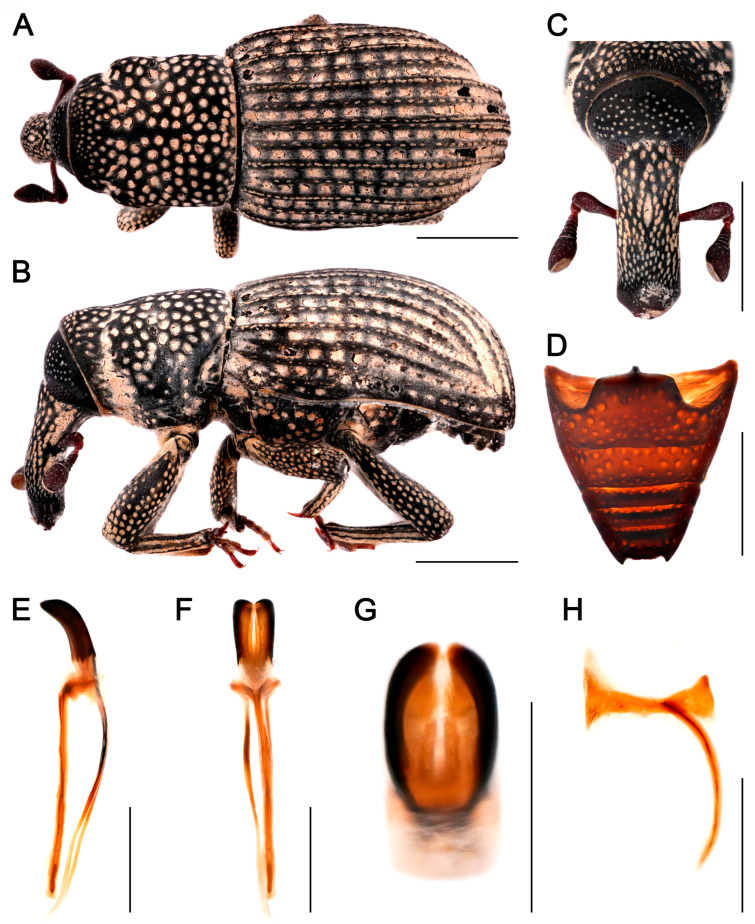
*Orthosinus sulcatus* sp. nov., holotype. (**A**) Dorsal habitus; (**B**) Lateral habitus; (**C**) Head, dorsal view; (**D**) Ventrites, ventral view; (**E**) Penis, lateral view; (**F**) Penis, ventral view; (**G**) Penis at apex, showing details of the pedon; (**H**) Spiculum gastrale. Scale bars: 1 mm (**A**–**D**); 0.5 mm (**E**–**H**).

**Figure 6:**
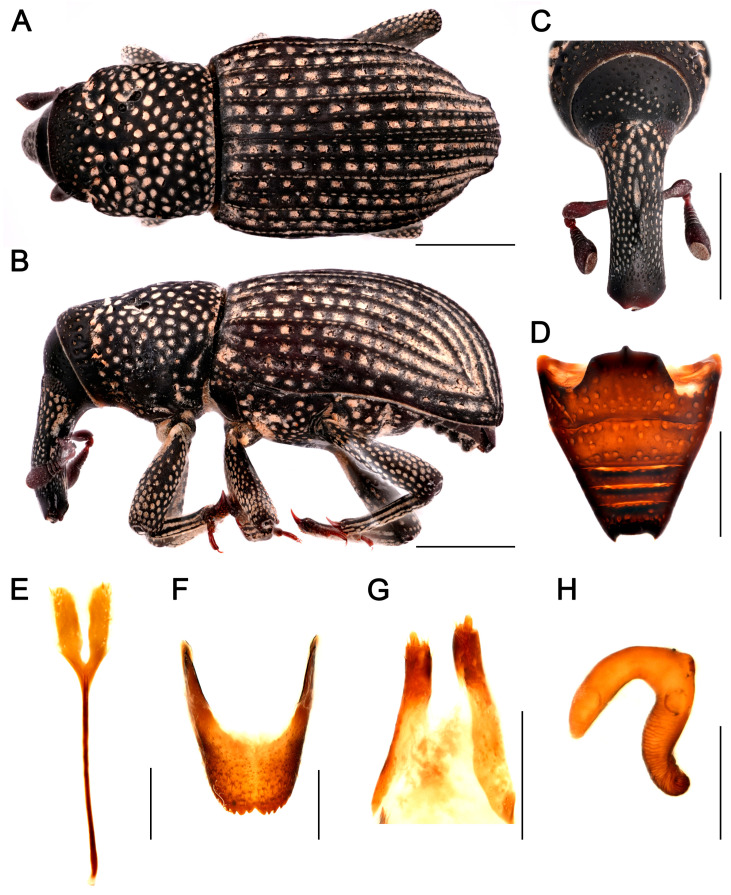
*Orthosinus sulcatus* sp. nov., paratype female (IOZ(E) 1965735). (**A**) Dorsal habitus; (**B**) Lateral habitus; (**C**) Head, dorsal view; (**D**) Ventrites, ventral view; (**E**) 8th sternite; (**F**) 8th tergite; (**G**) Ovipositor; (**H**) Spermatheca. Scale bars: 1 mm (**A**–**D**); 0.25 mm (**E**–**H**).

urn:lsid:zoobank.org:act:A999C28C-889F-447B-BDDA-C25DB92E7F3C
Figure 5 and Figure 6
Material examined. Holotype: 1♂, China, Sichuan Province, Chengdu City, Pengzhou City, Longmenshan Township, Jiufeng Village, Group 16, Dengdengshi, 31.340591° N, 103.883525° E, elev. 1362 m, 08/VIII/2018, Xiaoliang Yang leg. IOZ(E) 1965746. Paratypes: 10♂♂5♀♀, same data as holotype, IOZ(E) 1965734–1965745, IOZ(E) 1965747, IOZ(E) 1965749, IOZ(E) 1965750. 1♂, Sichuan Province, Chengdu City, Pengzhou City, Longmenshan Township, Zushi Hall, 31.326005° N, 103.85487° E, elev. 1902 m, 09/VIII/2018, Xiaoliang Yang leg. IOZ(E) 1965748.
Type locality. Dengdengshi, Group 16, Jiufeng Village, Longmenshan Township, Pengzhou City, Chengdu City, Sichuan Province, China.
**Comparative diagnosis.** *O. sulcatus* sp. nov. resembles *O. medogensis* but differs in the following characters: (i) head with small punctures, while large in *O. medogensis*; (ii) rostrum with coarse punctures, dorsally with a subrhomboidal sulcus at middle, versus rugose punctures, without subrhomboidal sulcus in *O. medogensis*; (iii) elytral lateral margin without arcuate tubercles, versus with small arcuate tubercles in *O. medogensis*; (iv) pedon apically thick, medially not connate in dorsal view, whereas thin, connate in *O. medogensis*; (v) spiculum gastrale with furcal arm broader at base than in *O. medogensis*; (vi) 8th sternite with apodeme 2.3 times length of lamina, lamina sides nearly straight (vs. 1.7 times, lamina sides curved in *O. medogensis*); (vii) styli approximately one-third width of gonocoxite apices (vs. one-half in *O. medogensis*); (viii) spermatheca with corpus longer than in *O. medogensis*.
**Description. *Coloration*** (Figure 5A,B). Body entirely black; antennal scape and funicle, club black; tarsomeres reddish brown.
***Head*** (Figure 5C). Subglobular, with dense, small punctures, with a shallow, transverse sulcus between frons and rostrum; eyes elongate-oval, widely separated ventrally; rostrum elongate (Rl/Rw 2.50), shorter than pronotum (Rl/Pl 0.73), slightly curved in lateral view, base thick, with short pubescence and coarse punctures from base to apical one-fourth, dorsally with a subrhomboidal sulcus at middle; antennae inserted at middle of rostrum; scape long (l/w 3.00), not reaching eyes, gradually broadening from base to apex; funicular segment 1 longer than wide, segment 2 funnel-shaped, length equal to combined length of segments 3 + 4, segments 3–6 transverse; club spindle-shaped (l/w 1.5).
***Pronotum*** (Figure 5A,B). Longer than wide (Pl/Pw 1.13), widest posterior to mid-length, apical one-fourth distinctly constricted, sides subparallel; disc slightly convex in lateral view, with dense, coarse punctures, distance between punctures smaller than puncture diameter, punctures sparser and smaller on constricted area than on lateral part; densely covered with short pubescence, anterior margin without pilose pustules; postocular lobes absent.
***Scutellum*.** Small, elongate-oval.
***Elytra*** (Figure 5A,B). Longer than wide (El/Ew 1.30), widest at middle, apical 1/6 distinctly constricted, sides rounded; disc slightly convex in lateral view; interstriae distinctly elevated, subequal in width, with dense, short pubescence; interstriae 3 and 5 with pubescence denser than on other interstriae; striae deep, as wide as interstriae; punctures elongate-oval, gradually diminishing apically, distance between punctures subequal to a puncture length, punctures with dense short pubescence.
***Abdomen*** (Figure 5D). Abdominal ventrites densely covered with coarse punctures, marginal punctures larger than on median area; 2nd ventrite with anterior margin slightly convex at middle, posterior margins of ventrites 2–4 rectilinear; 2nd ventrite 0.5 times length of 1st ventrite, 3rd ventrite as long as 4th ventrite, 5th ventrite 2.8 times as wide as long, deeply emarginate apically.
***Legs*** (Figure 5A,B). Densely covered with short pubescence; femora and tibiae with punctures; procoxae subconical, contiguous, mesocoxae narrowly separated; profemur more robust than mesofemur and metafemur; femora unarmed; profemur 3.3 times as long as wide; tibiae bearing single, long uncus; tarsi long, tarsomeres 1–3 obconical, ventrally with dense erect setae, onychium elongate; claws free, divergent.
***Male genitalia*** (Figure 5E–H). Pedon 0.3 times length of temones, evenly curved in lateral view, sides subparallel, base symmetrical, apex distinctly narrowed, medially not connate in dorsal view; temones slender, distinctly curved; manubrium long, shorter than temones, slightly curved, approximately 2.0 times as wide as temones; spiculum gastrale robust, evenly curved; basal plate bifurcate, basal arms opposed, each furcal arm basally broad, apices acute.
***Female*** (Figure 6A–H). Body larger than in male; rostrum longer and slenderer, smooth and shining; punctures on pronotum smaller than in male; antennae inserted slightly anterior to middle of rostrum. 8th sternite with apodeme 2.3 times length of lamina; lamina bifurcate at base, sides nearly straight, apex with setae; 8th tergite 1.3 times as long as wide, posterior margin serrate, mesally deeply emarginate; surface gradually more coarsely punctate towards apex, from apical one-half to apex densely setose. Gonocoxites cylindrical, apices with dense setae; styli short, cylindrical, width approximately one-third width of gonocoxite apices, apices with setae; spermatheca falciform, with curved, apically rounded cornu; corpus robust; ramus and collum weakly developed; other characters without distinct differences from male.
**Measurement (mm).** Holotype. Bl: 4.65. Rl: 1.25, Rw: 0.50. Pl: 1.71, Pw: 1.51. El: 2.70, Ew: 2.08. Male paratypes. (*n* = 11): Bl: 3.82–4.60 (4.22). Rl: 1.08–1.26 (1.16), Rw: 0.40–0.50 (0.46). Pl: 1.40–1.70 (1.55), Pw: 1.25–1.50 (1.27). El: 2.10–2.67 (2.44), Ew: 1.73–2.05 (1.88). Female paratypes. (*n* = 5): Bl: 3.70–4.85 (4.35). Rl: 1.10–1.40 (1.28), Rw: 0.40–0.50 (0.45). Pl: 1.40–1.80 (1.56), Pw: 1.15–1.60 (1.39). El: 2.10–2.80 (2.55), Ew: 1.64–2.10 (1.88).
**Distribution.** Known only from the type locality in Sichuan Province, China (Figure 10).
**Etymology.** The species name is a Latin masculine adjective *sulcatus*, referring to the rostrum bearing an oval sulcus dorsally. Variable.
**DNA barcode.**
AACTCTATATTTTTTTCTCGGAACTTGGGCAGGTATAATTGGAACCTCTCTAAGATTGTTAATTCGCTTAGAACTAGGAAATCCAGGTTCATTAATTGGAAATGATCAAATCTATAATACCATCGTTACAGCTCATGCTTTTATTATAATTTTCTTTATAGTTATACCAATTATAATTGGAGGATTTGGAAACTGATTAATTCCTCTTATATTAGGAGCTCCCGATATAGCTTTTCCACGACTCAATAATTTAAGATTTTGAATTCTCCCCCCCTCTTTAATTCTTCTAGCTTCAAGAAGAGCTATCGAAAGAGGGGCAGGCACAGGATGAACTGTTTATCCTCCGCTTTTCATCTAATACAGCACATAGAGGGGCGCCAGTTGATTTAGCCATCTTCAGCTTACATATAGCAGGAATTTCTTCCATTCTGGGAGCCATTAATTTTATTTCTACAGCTATTAATATACGACCAAGAGGAATATTCTCTGAACGTTTAACACTTTTTATTTGAGCAGTTAGAATTACAGCTCTTTTACTACTTCTATCTCTCCCAGTTCTTGCAGGAGCTATTACTATGCTACTCACTGACCGAAATATTAATACTTCCTTCTTTGACCCTACAGGAGGAGGAGATCCAATTCTTTACCAACATCTATTC (GenBank accession number: PZ092819).

#### 3.2.4. *Orthosinus tengchongensis* Lü & Zhang sp. nov.

**Figure 7:**
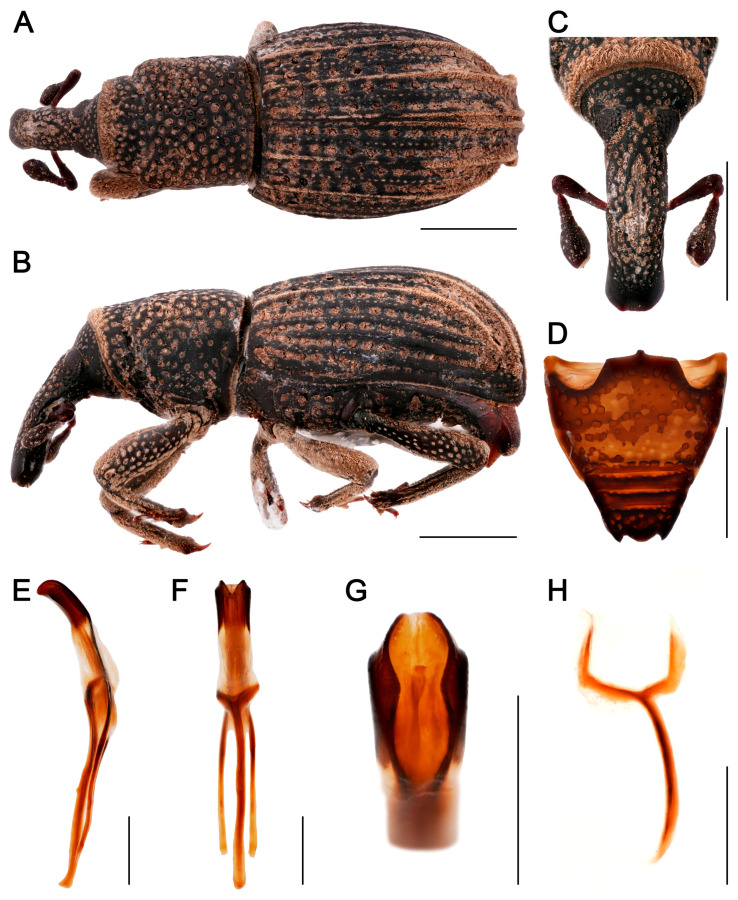
*Orthosinus tengchongensis* sp. nov., holotype. (**A**) Dorsal habitus; (**B**) Lateral habitus; (**C**) Head, dorsal view; (**D**) Ventrites, ventral view; (**E**) Penis, lateral view; (**F**) Penis, ventral view; (**G**) Penis at apex, showing details of the pedon; (**H**) Spiculum gastrale. Scale bars: 1 mm (**A**–**D**); 0.5 mm (**E**–**H**).

urn:lsid:zoobank.org:act:A6317B03-4BA1-4573-AFB0-5B1F69AF3386
Figure 7
Material examined. Holotype: 1♂, China, Yunnan Province, Tengchong City, Jietou Township, Datang Village, 25.73939° N, 98.69633° E, elev. 2010 m, 14/V/2006, Hongbin Liang leg. IOZ(E) 1965721. Paratype: 1♂, same data as holotype, IOZ(E) 1965722.
Type locality. Datang Village, Jietou Township, Tengchong City, Yunnan Province, China.
**Comparative diagnosis.** *O. tengchongensis* sp. nov. morphologically is most similar to *O. medogensis*, but distinguished by: (i) larger body (Bl: 4.20–4.40 mm, Bw: 1.83–2.03 mm vs. Bl: 3.50–4.40 mm, Bw: 1.50–1.80 mm in *O. medogensis*); (ii) rostrum with coarse punctures, versus rugose punctures in *O. medogensis*; (iii) pronotal sides subparallel, whereas rounded in *O. medogensis*; (iv) elytral interstriae 1 and 3 with pubescence projecting beyond outline apically, whereas in *O. medogensis* not projecting; (v) pedon 0.2 times length of temones, medially convex in dorsal view, manubrium slightly shorter than temones, approximately 1.6 times as wide as temones, while pedon 0.3 times length of temones, medially nearly straight in dorsal view, manubrium as long as temones, approximately 2.0 times as wide as temones in *O. medogensis*.
**Description. *Coloration*** (Figure 7A,B). Body entirely black; antennae and tarsomeres reddish brown.
***Head*** (Figure 7C). Subglobular, with dense, small punctures, with a shallow, transverse sulcus between frons and rostrum; eyes oval, widely separated ventrally; rostrum elongate (Rl/Rw 2.40), shorter than pronotum (Rl/Pl 0.73), slightly curved in lateral view, base thick, with short pubescence and coarse punctures from base to apical one-fourth, dorsally with short, indistinct longitudinal carina; antennae inserted at middle of rostrum; scape long (l/w 3.73), not reaching eyes, gradually broadening from base to apex; funicular segment 1 longer than wide, segment 2 funnel-shaped, length equal to combined length of segments 3 + 4, segments 3–6 transverse; club spindle-shaped (l/w 1.50).
***Pronotum*** (Figure 7A,B). Longer than wide (Pl/Pw 1.24), widest posterior to mid-length, apical one-fourth distinctly constricted, sides subparallel; disc slightly convex in lateral view, with dense, coarse punctures, distance between punctures smaller than puncture diameter, punctures sparser on constricted area than lateral part; densely covered with short pubescence, anterior margin with pilose pustules; postocular lobes absent.
***Scutellum.*** Small, subtriangular.
***Elytra*** (Figure 7A,B). Longer than wide (El/Ew 1.32), widest at middle, distinctly constricted subapically, sides rounded; disc nearly flat in lateral view; interstriae distinctly elevated, subequal in width, with dense, short pubescence; interstriae 1, 3 and 5 have more continuous pubescence, pubescence on interstriae 1, 3 and 5 distinctly denser than on other interstriae from apical one-third to apex; pubescence on interstriae 1 and 3 projecting beyond elytral outline apically; striae deep, as wide as interstriae; punctures elongate-oval, gradually diminishing apically, distance between punctures subequal to a puncture length, punctures with dense short pubescence.
***Abdomen*** (Figure 7D). Abdominal ventrites densely covered with coarse punctures, marginal punctures larger than on median area; 2nd ventrite with anterior margin slightly convex at middle, posterior margins of ventrites 2–4 rectilinear; 2nd ventrite 0.7 times length of 1st ventrite, 3rd ventrite as long as 4th ventrite, 5th ventrite 2.2 times as wide as long, deeply emarginate apically.
***Legs*** (Figure 7A,B). Densely covered with short pubescence; femora and tibiae with punctures; procoxae subconical, contiguous, mesocoxae narrowly separated; profemur more robust than mesofemur and metafemur; femora unarmed; profemur 2.6 times as long as wide; tibiae bearing single, long uncus; tarsi long, tarsomeres 1–3 obconical, ventrally with dense erect setae, onychium elongate; claws free, divergent.
***Male genitalia*** (Figure 7E–H). Pedon 0.2 times length of temones, evenly curved in lateral view, sides subparallel, base symmetrical, apex distinctly narrowed, medially convex in dorsal view; temones slender, distinctly curved; manubrium long, shorter than temones, slightly curved at basal 1/3, approximately 1.6 times as wide as temones; spiculum gastrale robust, slightly curved; basal plate bifurcate, basal arms opposed, apices acute.
**Female.** unknown.
**Measurement (mm).** Holotype. Bl: 4.40. Rl: 1.20, Rw: 0.50. Pl: 1.64, Pw: 1.32. El: 2.68, Ew: 2.03. Male paratype. Bl: 4.20. Rl: 1.06, Rw: 0.47. Pl: 1.58, Pw: 1.30. El: 2.46, Ew: 1.83.
**Distribution.** Known only from the type locality in Yunnan Province, China (Figure 10).
**Etymology.** This species is named after its type locality, Tengchong City. Adjective, variable.

#### 3.2.5. *Orthosinus urceolatus* Lü & Zhang sp. nov.

**Figure 8:**
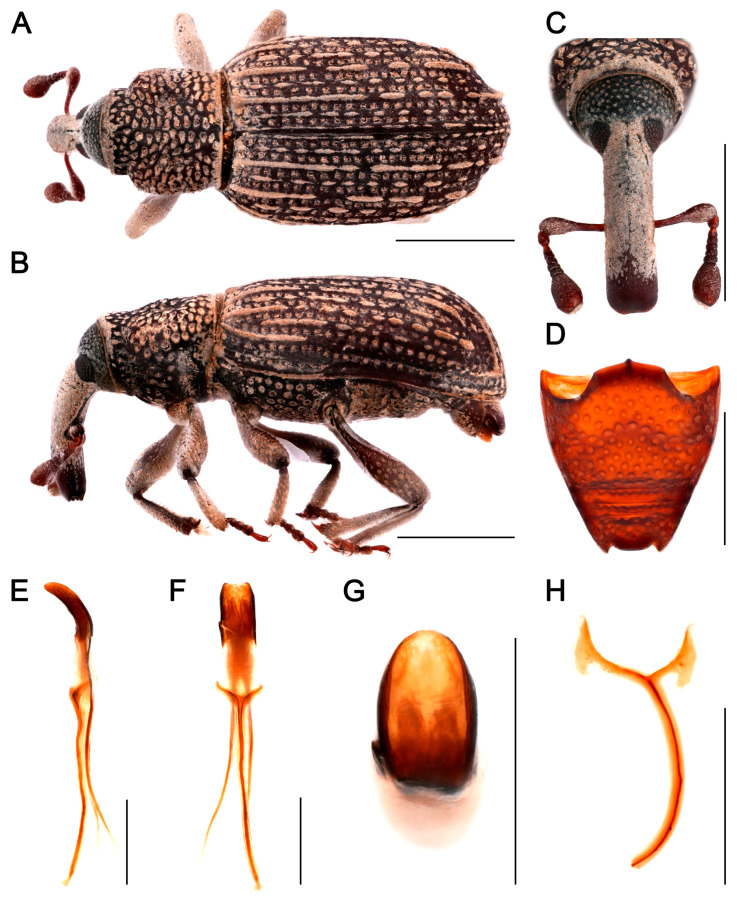
*Orthosinus urceolatus* sp. nov., holotype. (**A**) Dorsal habitus; (**B**) Lateral habitus; (**C**) Head, dorsal view; (**D**) Ventrites, ventral view; (**E**) Penis, lateral view; (**F**) Penis, ventral view; (**G**) Penis at apex, showing details of the pedon; (**H**) Spiculum gastrale. Scale bars: 1 mm (**A**–**D**); 0.5 mm (**E**–**H**).

**Figure 9:**
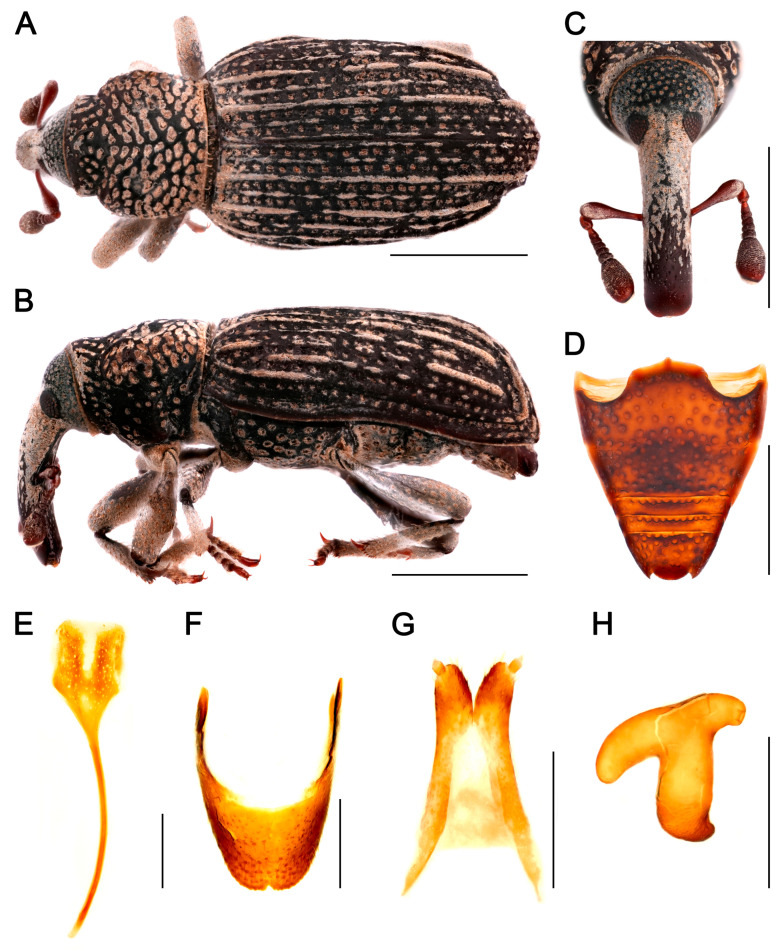
*Orthosinus urceolatus* sp. nov., paratype female (IOZ(E) 1965767). (**A**) Dorsal habitus; (**B**) Lateral habitus; (**C**) Head, dorsal view; (**D**) Ventrites, ventral view; (**E**) 8th sternite; (**F**) 8th tergite; (**G**) Ovipositor; (**H**) Spermatheca. Scale bars: 1 mm (**A**–**D**); 0.25 mm (**E**–**H**).

**Figure 10:**
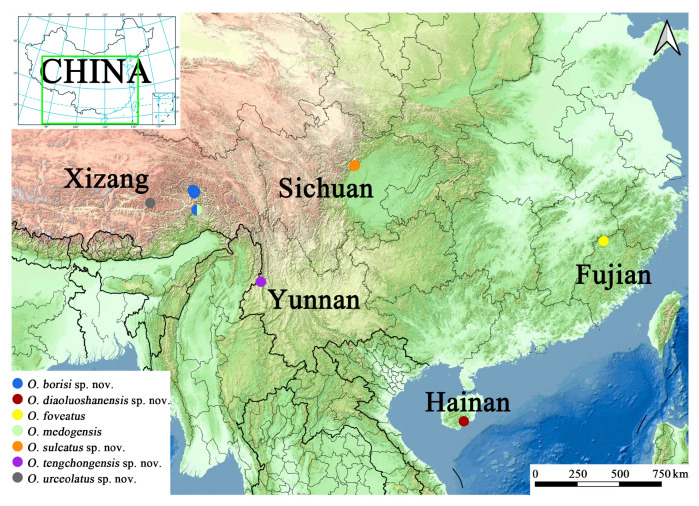
Distribution of *Orthosinus* species in China.

urn:lsid:zoobank.org:act:DD676314-D442-4823-8D1C-19EF8CABD658
Figure 8 and Figure 9
Material examined. Holotype: 1♂, China, Xizang, Linzhi City, Bayi District, G318 Highway (near Sejila Mountain Pass), 29.56165° N, 92.57520° E, elev. 3852 m, 29/VII/2018, Run Zhou leg. IOZ(E) 1965760. Paratypes: 5♂♂9♀♀, same data as holotype, IOZ(E) 1965751–1965756, IOZ(E) 1965758, IOZ(E) 1965759, IOZ(E) 1965761–1965764, IOZ(E) 1965766, IOZ(E) 1965767.
Type locality. G318 Highway (near Sejila Mountain Pass), Bayi District, Linzhi City, Xizang Autonomous Region, China.
**Comparative diagnosis.** *O. urceolatus* sp. nov. is distinctive in having an urceolate pronotum, a character unique within the genus. It is most similar to *O. himalayanus* (Marshall, 1931) in general morphology, but differs in the following characters: (i) body wider (Bw: 1.55–1.80 mm vs. 1.20–1.50 mm in *O. himalayanus*); (ii) eyes larger, longer than shortest side of club (vs. as long in *O. himalayanus*); (iii) rostrum shorter than pronotum in both sexes (vs. female as long, male shorter in *O. himalayanus*); (iv) antennal funicular segment 2 shorter than combined length of segments 3 + 4 (vs. equal in *O. himalayanus*); (v) elytra widest at middle (vs. basal one-third in *O. himalayanus*).
**Description. *Coloration*** (Figure 8A,B). Body entirely black; rostral apex, pronotum, and elytra dark reddish brown; antennae and tarsomeres reddish brown.
***Head*** (Figure 8C). Subglobular, with dense, small punctures, with a shallow, transverse sulcus between frons and rostrum; eyes oval, widely separated ventrally; rostrum elongate (Rl/Rw 2.89), longer than pronotum (Rl/Pl 1.05), curved in lateral view, base thick, with dense, short pubescence and coarse punctures from base to apical one-fourth; antennae inserted anterior to rostral mid-length; scape long (l/w 3.13), not reaching eyes, gradually broadening from base to apex; funicular segment 1 longer than wide, segment 2 funnel-shaped, length shorter than combined length of segments 3 + 4, segments 3–6 transverse; club spindle-shaped (l/w 1.50).
***Pronotum*** (Figure 8A,B). Shorter than wide (Pl/Pw 0.95), widest at middle, apical one-fifth distinctly constricted, gradually narrowed from apical one-third to base; disc slightly convex in lateral view, with dense, coarse punctures, distance between punctures smaller than puncture diameter, punctures sparser and smaller on constricted area than on lateral part; densely covered with short pubescence, apical with pilose pustules; with one broad oval sulcus on each side of the middle; postocular lobes absent.
***Scutellum.*** Small, elongate-oval.
***Elytra*** (Figure 8A,B). Longer than wide (El/Ew 1.46), widest at middle, apical one-seventh slightly constricted, sides rounded; disc flat in lateral view; interstriae slightly elevated, subequal in width, with dense, short pubescence; interstriae 3 and 5 with pubescence denser than on other interstriae, forming continuous pilose pustules; striae deep, as wide as interstriae; punctures rounded, distance between punctures subequal to a puncture diameter, punctures with dense, short pubescence.
***Abdomen*** (Figure 8D). Abdominal ventrites densely covered with coarse punctures, marginal punctures larger than on median area; 2nd ventrite with anterior margin slightly convex at middle, posterior margins of ventrites 2–4 rectilinear; 2nd ventrite 0.8 times length of 1st ventrite, 3rd ventrite as long as 4th ventrite, 5th ventrite 2.7 times as wide as long, deeply emarginate apically.
***Legs*** (Figure 8A,B). Densely covered with short pubescence; femora and tibiae with punctures; procoxae subconical, contiguous; mesocoxae narrowly separated; profemur more robust than mesofemur and metafemur; femora unarmed; profemur 2.8 times as long as wide; tibiae bearing single, long uncus; tarsi long, tarsomeres 1–3 obconical, ventrally with dense erect setae, onychium elongate; claws free, divergent.
***Male genitalia*** (Figure 8E–H). Pedon 0.3 times length of temones, evenly curved in lateral view, sides subparallel, base symmetrical, apex distinctly narrowed; temones slender, distinctly curved; manubrium long, as long as temones, distinctly curved from base to apex, approximately 1.5 times as wide as temones; spiculum gastrale robust, evenly curved; basal plate bifurcate, basal arms opposed, upper part of each basal arm approximately triangular, apices with rounded angles.
***Female*** (Figure 9A–H). Body larger than in male, some individuals with body coloration darker than in male, pronotum and elytra black; rostrum longer and slenderer, smooth and shining; antennae inserted slightly anterior to middle of rostrum; abdominal ventrites narrower than in male. 8th sternite with apodeme 2.5 times length of lamina; lamina bifurcate at base, sides nearly straight, apex with setae; 8th tergite 1.5 times as long as wide, posterior margin serrate, mesally deeply emarginate; surface gradually more coarsely punctate towards apex, from apical one-half to apex densely setose. Gonocoxites cylindrical, apices with dense setae; styli short, cylindrical, width approximately one-third width of gonocoxite apices, apices with setae; spermatheca falciform, with curved, apically rounded cornu; corpus very robust; ramus and collum developed; other characters without distinct differences from male.
**Measurement (mm).** Holotype. Bl: 3.78. Rl: 1.10, Rw: 0.38. Pl: 1.05, Pw: 1.10. El: 2.40, Ew: 1.64. Male paratypes. (*n* = 5): Bl: 3.45–4.00 (3.76). Rl: 1.05–1.15 (1.07), Rw: 0.34–0.40 (0.39). Pl: 1.04–1.10 (1.06), Pw: 1.04–1.10 (1.08). El: 2.20–2.60 (2.45), Ew: 1.58–1.70 (1.63). Female paratypes. (*n* = 9): Bl: 3.50–4.10 (3.78). Rl: 1.08–1.25 (1.13), Rw: 0.30–0.38 (0.34). Pl: 1.00–1.15 (1.08), Pw: 1.05–1.20 (1.11). El: 2.30–2.70 (2.42), Ew: 1.55–1.80 (1.61).
**Distribution.** Known only from the type locality in Xizang Autonomous Region, China (Figure 10).
**Etymology.** The species name is a Latin masculine adjective *urceolatus*, referring to the pronotum with an urceolate shape. Variable.
**DNA barcode.**
TACCCTTTATTTTATTTTCGGGACTTGATCTGGTATAATCGGAACATCCCTAAGAATGCTTATTCGTATAGAACTAGGAAGACCAGGCTCTCTAATTGGAAATGATCAAATTTACAACACTATTGTCACAGCCCATGCCTTCATTATAATTTTTTTTATAGTTATACCCATTATGATTGGCGGCTTCGGTAATTGATTAGTACCTTTAATATTAGGAGCTCCTGATATAGCCTTTCCACGACTTAATAATATAAGATTTTGGCTCCTTCCCCCCTCCTTAATCTTACTTATTATAAGAAGAATTATTGAAAGGGGGGCAGGAACTGGATGAACAGTTTATCCTCCACTTTCATCAAACACAGCCCATAGCGGAGCTCCTGTAGATCTAGCTATTTTCAGGCTTCACATAGCAGGAATTTCCTCCATTCTAGGAGCTATTAATTTTATTTCTACAGCCATCAATATACGTCCAAGAGGTATATTTTCTGAACGTTTATCTCTATTCATTTGAGCAGTAGCAATCACAGCCCTCCTCCTACTACTTTCCCTCCCAGTTTTAGCAGGAGCAATCACCATACTTTTAACCGATCGAAATATTAATACCTCATTCTTTGATCCTACAGGAGGGGGAGACCCAATTCTCTATCAACACCTATTC (GenBank accession number: PZ092820).

#### 3.2.6. Key to Species of the Genus *Orthosinus* in China

1. Rostrum dorsally without a subrhomboidal sulcus at middle.................................................... 2
– Rostrum dorsally with a subrhomboidal sulcus at middle.
Additional characters: Rostrum shorter than pronotum. Pronotum anterior margin without pilose pustules. Elytra without interrupted pilose pustules; interstrial pubescence not fasciculate. Pedon medially not connate in dorsal view. Body length 3.70–4.85 mm, width 1.25–2.10 mm.............................................................................................................................. *O. sulcatus* sp. nov.
2. Rostrum shorter than or as long as pronotum................................................................................ 3
– Rostrum longer than pronotum.......................................................................................................... 6
3. Pronotum widest at middle................................................................................................................ 4
– Pronotum widest at apical one-third.
Additional characters: Rostrum as long as pronotum, with fine punctures. Antennal club 2.0 times as long as wide. Elytra with arcuate tubercles laterally. Body length 3.50 mm............................................................................................................................................. *O. foveatus*
4. Pronotum longer than wide.............................................................................................................. 5
– Pronotum as long as wide.
Additional characters: Pronotum with very coarse punctures, anterior margin without pilose pustules. Rostrum dorsally with indistinct longitudinal carina. Elytra with small arcuate tubercles laterally; disc distinctly convex in lateral view. Body length 3.60 mm, width 1.85 mm.................................................................. *O. diaoluoshanensis* sp. nov.
5. Rostrum with coarse punctures. Pronotal sides subparallel. Elytral interstriae 1 and 3 with pubescence projecting beyond outline apically. Pedon 0.2 times length of temones, medially convex in dorsal view; manubrium slightly shorter than temones, approximately 1.6 times as wide as temones. Body length 4.20–4.40 mm, width 1.83–2.03 mm................................................................................................................. *O. tengchongensis* sp. nov.
– Rostrum with rugose punctures from base to apical one-third. Pronotal sides rounded. Pubescence on interstriae 1 and 3 not projecting beyond outline. Pedon 0.3 times length of temones, medially nearly straight in dorsal view; manubrium as long as temones, approximately 2.0 times as wide as temones. Body length 3.50–4.40 mm, width 1.50–1.80 mm........................................................................................................................... *O. medogensis*
6. Antennal scape 5.00 times as long as wide; club 1.82 times as long as wide. Pronotal punctures round, with longitudinal pilose pustules medially. Pilose pustules on elytral interstriae broad, some wider than interstriae. Pedon gradually broadening from base to apical one-third, apex thin in lateral view. Female 8th sternite with apodeme straight, 1.4 times length of lamina, lamina sides slightly curved. 8th tergite 0.9 times as long as wide, posterior margin nearly smooth. Spermatheca with ramus and collum weakly developed. Body length 3.50–4.30 mm, width 1.65–2.20 mm…………………………………………………………….. *O. borisi* sp. nov.
– Antennal scape 3.13 times as long as wide; club 1.50 times as long as wide. Pronotal punctures elongate-oval, without pilose pustules. Pilose pustules on elytral interstriae narrow, usually narrower than interstriae. Pedon sides subparallel, apex not thin. Female 8th sternite with apodeme curved, 2.5 times length of lamina, lamina sides nearly straight. 8th tergite 1.5 times as long as wide, posterior margin serrate. Spermatheca with ramus and collum developed. Body length 3.45–4.10 mm, width 1.55–1.80 mm......................................... *O. urceolatus* sp. nov.

## 4. Discussion

This study revises the taxonomic knowledge of *Orthosinus* in China. Prior to this work, only *O. foveatus* and *O. medogensis* were recorded from the country. This pattern is consistent with the current understanding of Chinese Stromboscerini, which is still largely based on scattered taxonomic treatments of individual genera [4,7,9]. Previous studies have shown that most Chinese representatives of Stromboscerini are recorded from southern and southwestern montane regions, suggesting that the tribe remains substantially under-sampled in these biodiversity-rich areas [4,7,9,21,23]. The addition of five new species more than triples the known Chinese fauna to seven. Considering that extensive areas of southern China remain poorly sampled for Stromboscerini, particularly the mountainous regions of Yunnan, western Sichuan, and southeastern Xizang, further undescribed diversity almost certainly awaits discovery.

The geographic origins of the new species significantly expand the documented range of the genus in China. In particular, *O. diaoluoshanensis* sp. nov. from Hainan Island represents the first record of the genus from a Chinese offshore island. The remaining new species all originate from areas adjacent to the eastern Himalayas and the Hengduan Mountains. The type locality of *O. urceolatus* sp. nov., near the Sejila Mountain Pass at an elevation of 3852 m, is particularly noteworthy, as it represents one of the highest confirmed elevation records for *Orthosinus* currently available from China and possibly across the known range of the genus. This suggests that *Orthosinus* may inhabit a broader elevational range than previously assumed.

Morphologically, the five new species are distinguished by several characters that are taxonomically informative for species-level diagnosis in *Orthosinus*. The most striking character is the urceolate pronotum of *O. urceolatus* sp. nov., which is unique within the genus and readily separates this species from all known congeners. *O. borisi* sp. nov. is characterized by dense pilose pustules on both the pronotum and elytra, traits that are shared to varying degrees with the geographically proximate *O. medogensis* but are far more pronounced in the new species. *O. sulcatus* sp. nov. is the only Chinese species bearing a distinct subrhomboidal sulcus on the dorsal surface of the rostrum, a diagnostic feature that is immediately recognizable. *O. diaoluoshanensis* sp. nov. can be separated from the morphologically similar *O. foveatus* by the absence of longitudinal rugae on the pronotum and differences in body proportions, while *O. tengchongensis* sp. nov. is most reliably distinguished from *O. medogensis* by the projecting pubescence on elytral interstriae 1 and 3 and distinct male genital characters.

DNA extraction was attempted for additional specimens, but amplification failed in most cases due to DNA degradation associated with long-term preservation, specimen age, and limited destructive sampling permitted for rare type material. Successful *COI* amplification was obtained only from freshly collected specimens of *O. sulcatus* sp. nov. and *O. urceolatus* sp. nov. The molecular analyses, although limited in taxonomic scope, provide an informative complement to the morphological evidence. Both the ABGD and jMOTU species delimitation methods consistently partitioned *O. urceolatus* sp. nov. and *O. sulcatus* sp. nov. as distinct MOTUs, independently corroborating their morphological separation. In the Maximum Likelihood tree, the two species formed a moderately supported clade (bootstrap = 32) within *Orthosinus*. The relatively low nodal support is likely attributable to the small taxon sampling of *COI* sequences currently available for this genus, a limitation that will be addressed as additional sequences are generated in ongoing studies. The phylogenetic position of *Orthosinus* as sister to the genus *Tasactes*, with strong support (bootstrap = 100), is consistent with earlier findings by Grebennikov [19].

Several limitations of the present study should be acknowledged. First, molecular data were obtained for only two of the five new species, a constraint dictated by the availability of specimens suitable for DNA extraction among older museum specimens. The taxonomic status of *O. borisi* sp. nov., *O. diaoluoshanensis* sp. nov., and *O. tengchongensis* sp. nov. therefore rests solely on morphological evidence, and their independent species status will benefit from future molecular validation. Second, females remain unknown for *O. diaoluoshanensis* sp. nov. and *O. tengchongensis* sp. nov. The absence of female specimens limits the completeness of the morphological diagnoses and prevents evaluation of potential sexual dimorphism in these species. Third, the sampling effort across China remains geographically uneven. Large portions of south-central and southwestern China have yet to be systematically surveyed for leaf litter weevils. Future collecting efforts should prioritise these under-sampled areas, and the inclusion of freshly collected material suitable for DNA extraction will be essential for building a more complete molecular framework for the genus. Despite these limitations, this study lays important groundwork. The morphological descriptions, identification key, and distribution map provide practical tools for recognizing the Chinese species of *Orthosinus*. As barcode reference libraries for Stromboscerini continue to grow, species delimitation analyses incorporating broader geographic and taxonomic sampling will allow for more robust testing of species boundaries and phylogenetic relationships within this poorly understood group.

## Data Availability

The original contributions presented in this study are included in the article. Further inquiries can be directed to the corresponding author.
